# Supplementary material for: MetaRibo-Seq measures translation in microbiomes
Source: Nat Commun. 2020 Jun 29;11:3268. doi: 10.1038/s41467-020-17081-z (PMC7324362; doi:10.1038/s41467-020-17081-z)
Supplement: Supplementary file 10 — Supplementary Data 7 [file 41467_2020_17081_MOESM10_ESM.zip › File2/Confidence_High_Taxonomy/922_out.krona.html]

Javascript must be enabled to view this page.

members
magnitude
magnitudeUnassigned
count
unassigned
taxon
rank

922\_out

43


SRS042131\_contig\_number\_6516
superkingdom
1
43
2

1224
2
phylum

class
28216
1

80840
1
order

1
80864
family

genus
219181
1

species
1
1658672

SRS015158\_contig\_number\_13029

1236
1
class

order
91347
1

1
543
family

genus
413496
1

species
28141

SRS1055066\_contig\_number\_2603
1

23
74201
phylum

class
23
203494

23
48461
order

1647988
23
family

genus
239934
23

1638783

SRS021484\_contig\_number\_2033
1
species

239935

SRS011239\_contig\_number\_341SRS011302\_contig\_number\_1208SRS011405\_contig\_number\_1990SRS014613\_contig\_number\_45SRS015890\_contig\_number\_2468SRS018817\_contig\_number\_9219SRS019601\_contig\_number\_243SRS023914\_contig\_number\_783SRS048870\_contig\_number\_859SRS076976\_contig\_number\_1643SRS098644\_contig\_number\_2480SRS098881\_contig\_number\_1884SRS101433\_contig\_number\_4430SRS1041129\_contig\_number\_2SRS1055049\_contig\_number\_38SRS1055067\_contig\_number\_28SRS1055076\_contig\_number\_35SRS1055099\_contig\_number\_68SRS143417\_contig\_number\_761SRS143876\_contig\_number\_12070SRS144753\_contig\_number\_828SRS971276\_contig\_number\_685
22
species

1
976
phylum

class
117743
1

200644
1
order

1
49546
family

1
1016

SRS051244\_contig\_number\_13593
genus

phylum
1
256845

1
1313211
class

order
278082
1

species
1
2094242

SRS100907\_contig\_number\_203

1239
5
phylum

class
1
186801

186802
1
order

family
186803
1

43994
1
genus


SRS015215\_contig\_number\_2047
43995
1
species

class
4
91061

order
4
186826

186828
1
family

genus
1
117563

1
46124

SRS016200\_contig\_number\_1089
species

family
2
1300

1301
2
genus

1579341

SRS142791\_contig\_number\_1602
1
species

species
1579343

SRS013164\_contig\_number\_1996
1

186827
1
family

1
46123
genus

species
46125

SRS043755\_contig\_number\_77
1

10
32066
phylum

class
10
203490

order
203491
10

1
203492
family

1
848
genus


SRS013949\_contig\_number\_17398
860
1
species

family
1129771
9

1
168808
genus


SRS011111\_contig\_number\_contig-100\_3618.3618
187101
1
species

32067
8
genus

5
712359

SRS011115\_contig\_number\_contig-100\_97.59697.59697SRS012279\_contig\_number\_contig-100\_18.31259SRS012983\_contig\_number\_contig-100\_4052.32130SRS014404\_contig\_number\_contig-100\_652.653SRS014684\_contig\_number\_8798
species

species
157688

SRS011098\_contig\_number\_8064
1

species
712357

SRS012922\_contig\_number\_contig-100\_2180.2180.2180SRS013663\_contig\_number\_9450
2
